# Brain and Adrenal Metastasis From Unknown Primary Tumor: A Case Report

**DOI:** 10.7759/cureus.26438

**Published:** 2022-06-29

**Authors:** Ryo Katsumata, Yasumasa Monobe, Akihisa Akagi, Tomoki Yamatsuji, Yoshio Naomoto

**Affiliations:** 1 Department of Health Care Medicine, Kawasaki Medical School General Medical Center, Okayama, JPN; 2 Department of Pathology, Kawasaki Medical School General Medical Center, Okayama, JPN; 3 Department of General Surgery, Kawasaki Medical School General Medical Center, Okayama, JPN

**Keywords:** stereotactic radiotherapy, cancer of unknown primary, carcinoembryonic antigen, adrenal metastasis of unknown primary, brain metastasis of unknown primary

## Abstract

The clinical management of brain metastasis (BM) and adrenal metastasis (AM) of cancer of unknown primary (CUP) can be challenging. A 73-year-old man presented to the hospital with sudden-onset hemiplegia. His laboratory data were normal, except for elevated levels of carcinoembryonic antigen (CEA) (33.8 ng/mL). Contrast-enhanced magnetic resonance imaging revealed a 2-cm mass with ring enhancement in the right parietal lobe and extensive vasogenic edema around the tumor. The lesion was diagnosed as BM; however, we could not detect the primary origin by fluorodeoxyglucose (FDG) positron emission tomography-computed tomography (PET-CT). Stereotactic radiotherapy was then administered, resulting in reduced tumor size and relief of symptoms. Follow-up after one year revealed an elevated CEA level (148.6 ng/mL) and remarkable fluorodeoxyglucose (FDG) uptake in the right adrenal gland, with an area of enhancement of 20 mm, on FDG-positron emission tomography computed tomography, with normal findings in other distant organs. He underwent adrenalectomy, and the adrenal tumor was diagnosed as a poorly differentiated adenocarcinoma likely of lung origin based on the histopathologic and immunohistochemistry findings of cytokeratin (CK) 7 (+), CK 20 (-), thyroid transcription factor-1 (TTF-1) (+), inhibin (-), napsin A (+), prostate-specific antigen (PSA) (-), caudal type homeobox 2 (CDX-2) (-), synaptophysin (-), and p40 (-). Metastatic tumors of unknown primary origin remain latent. Aggressive treatment of these lesions can be beneficial for symptom relief, diagnosis, and prolongation of survival.

## Introduction

Brain metastasis (BM) with a primary original tumor is known as BM of cancer of unknown primary (CUP), with a reported prevalence of up to 15% [1]. Previous studies have described the characteristics of BM-CUP, including a relatively poor prognosis (median survival between three and 12 months), a wide variety of clinical symptoms, and data on the number of lesions and their sites [2,3]. Despite the development and availability of effective imaging technologies such as positron emission tomography-computed tomography (PET-CT), some tumors are still diagnosed as BM-CUP. Treatment should be initiated on diagnosis and the primary site should be identified. The treatment options considered are surgery, radiosurgery, radiotherapy, and/or systemic medical therapies [4].

Although the adrenal glands are a common site of malignant tumor metastasis, adrenal metastasis (AM) without a detectable primary tumor is rare. Generally, primary carcinoma is recognized by the time suspected adrenal metastases are discovered. The frequency of unknown primary cancers presenting as adrenal masses is reportedly 0.2% [5]. Only a few case reports have been published regarding AM-CUP [6,7]. These patients presented with metastatic lesions only in the adrenal gland. Due to the rarity of the condition and insufficient clinical data, treatment strategies for AM-CUP have not been well established. Here, we report a case of BM and AM from an unknown primary tumor in a patient who showed a positive response to radiotherapy and surgical treatment.

## Case presentation

The patient was a 73-year-old man with a medical history of surgical treatment for esophageal cancer (15 years prior, squamous cell carcinoma, pT1 {LPM} N0 M0, pathological stage 0) and colon cancer (five years prior, adenocarcinoma, pT1 {SM} N0 M0, pathological stage 1, immunohistochemistry staining: cytokeratin {CK} 7 {-}, CK 20 {+}, thyroid transcription factor-1 {TTF-1} {-}, and caudal type homeobox 2 {CDX-2} {+}). He had no medical history of lung disease. He was a past smoker (20 cigarettes per day for 40 years) and a non-drinker. 

**Figure 1:**
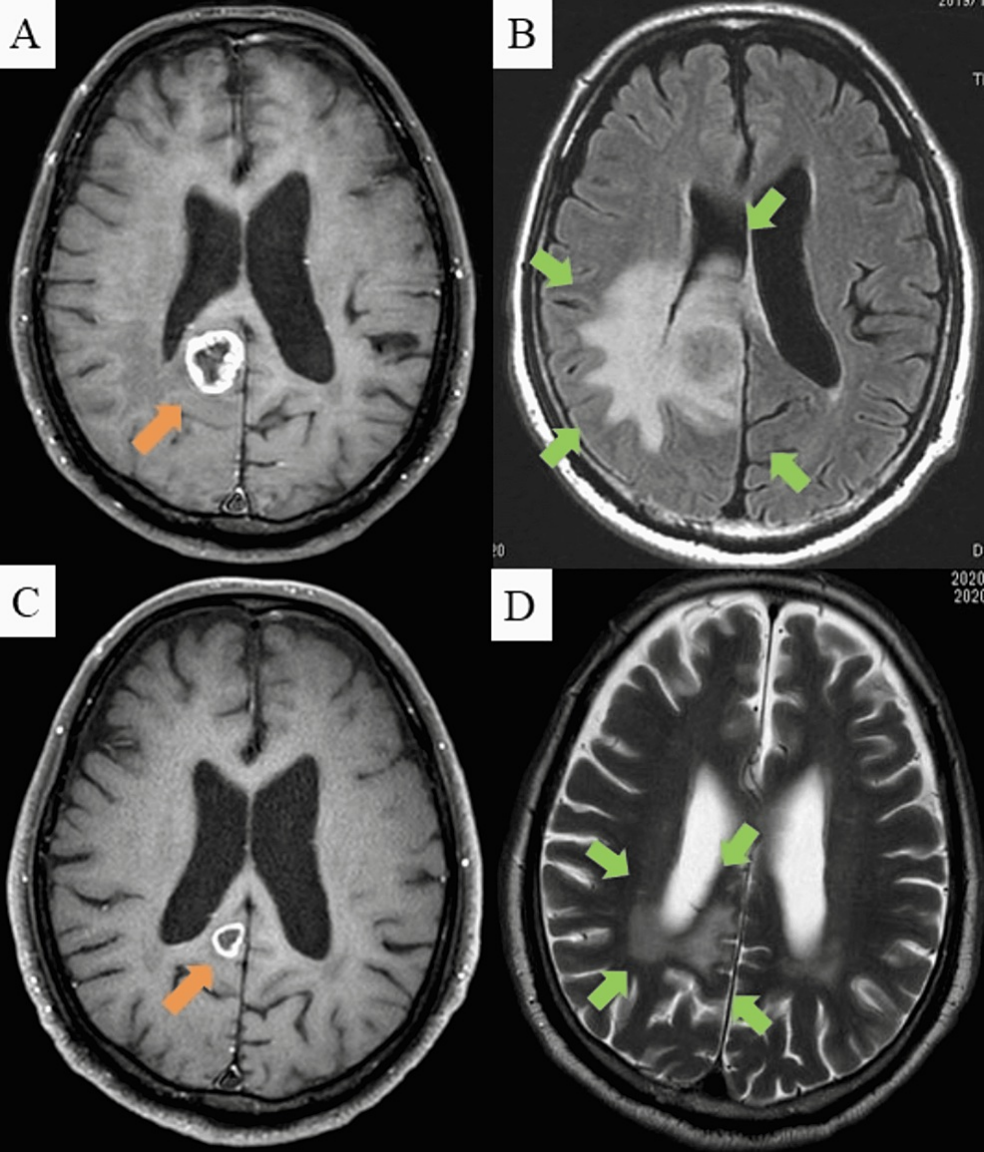
Magnetic resonance image of brain. (A) Contrast-enhanced magnetic resonance image revealing a 2-cm mass with ring enhancement in the right parietal lobe in the post-contrast T1-weighted condition (orange arrow). (B) Extensive vasogenic edema (green arrows) was confirmed by fluid-attenuated inversion recovery. (C) Reduced tumor with ring enhancement (orange arrow) and (D) reduced vasogenic edema (green arrows) after radiotherapy.

He visited a hospital emergency department due to sudden-onset hemiplegia. His laboratory data were normal, except for elevated levels of carcinoembryonic antigen (CEA) (33.8 ng/mL). Contrast-enhanced magnetic resonance imaging (MRI) showed a 2-cm mass with ring enhancement in the right parietal lobe in the post-contrast condition. Fluid-attenuated inversion recovery confirmed extensive vasogenic edema (Figure 1, panels A and B).

**Figure 2:**
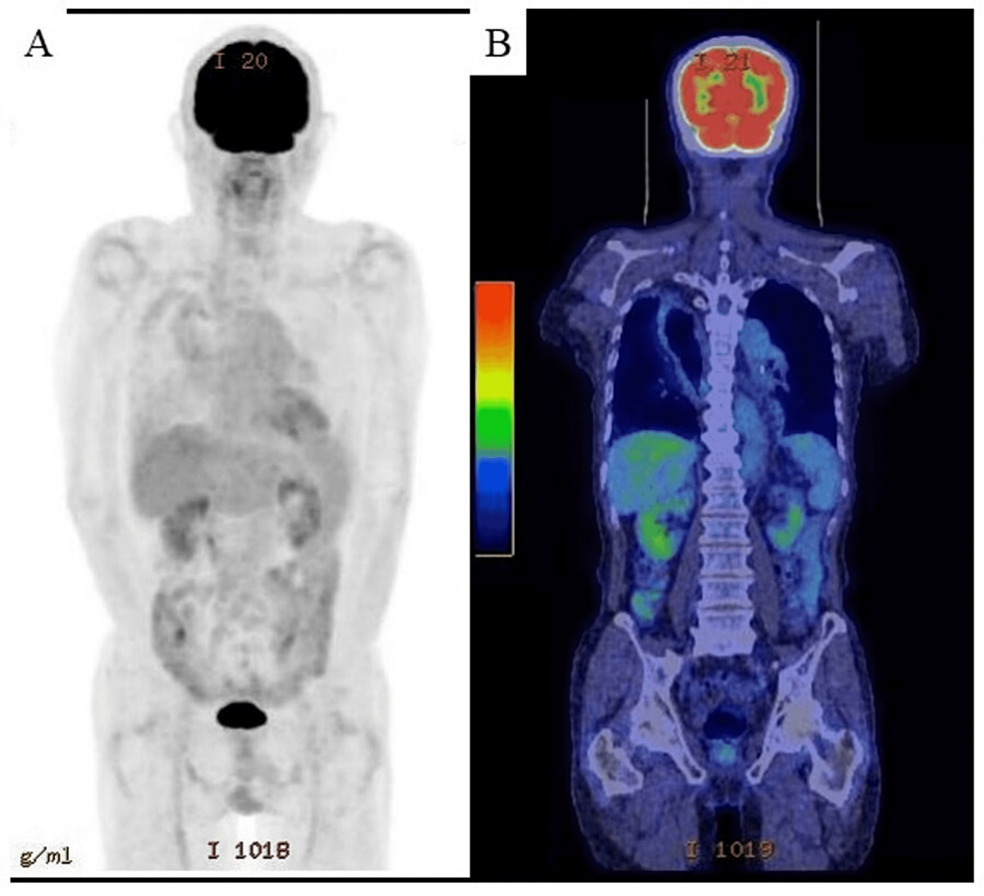
FDG positron emission tomography computed tomography at the time when brain tumor was detected. (A and B) The images show unremarkable FDG uptake in distant organs from the brain. FDG: fluorodeoxyglucose

Based on imaging findings, the patient was diagnosed with BM; however, no primary tumor was found by contrast-enhanced cervical, chest, and abdominal CT, and referred to our hospital for further management. At our institution, fluorodeoxyglucose (FDG) PET-CT showed unremarkable FDG uptake in distant organs including thyroid gland, lung, colon, liver, adrenal gland, kidney, and prostate (Figure 2, panels A and B).

Gastrointestinal endoscopy revealed no malignant lesions in the esophagus, stomach, duodenum, or colon. We diagnosed the brain tumor as BM-CUP, and stereotactic radiotherapy for BM-CUP (7 Gy × 4) was conducted after a multidisciplinary expert team meeting. We did not perform a brain biopsy. The tumor reduced in size after treatment and the patient’s neurological symptoms were relieved (Figure 1, panels C and D).

**Figure 3:**
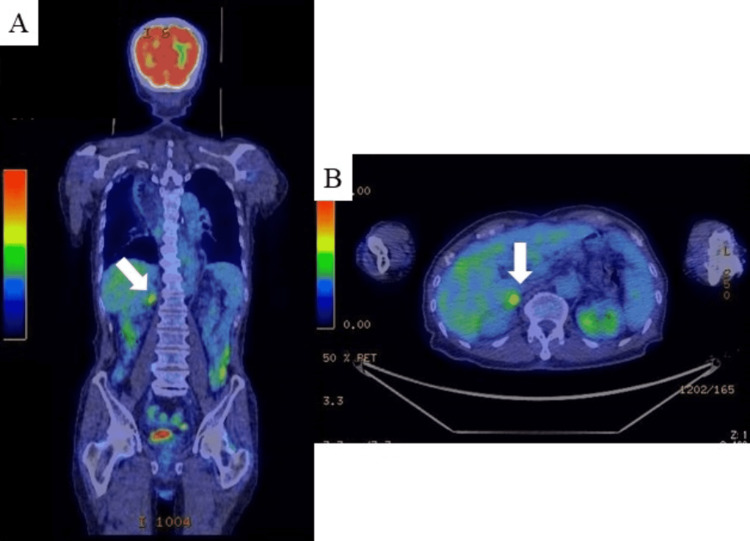
FDG positron emission tomography computed tomography after one-year surveillance period. (A and B) The images show remarkable FDG uptake in the right adrenal gland with an enlargement area of 20 mm (white arrows). FDG: fluorodeoxyglucose

Over one-year regular surveillance period, an elevated CEA level (148.6 ng/mL) was observed without any symptoms. FDG PET-CT revealed remarkable FDG uptake in the right adrenal gland with an area of enhancement of 20 mm and maximum standardized uptake value of 4.29 and 4.28 in the early and delayed phases, respectively, but not in any other distant organ (Figure 3, panels A and B). No malignant lesions were confirmed on upper and lower gastrointestinal endoscopy or thyroid ultrasound tests.

**Figure 4:**
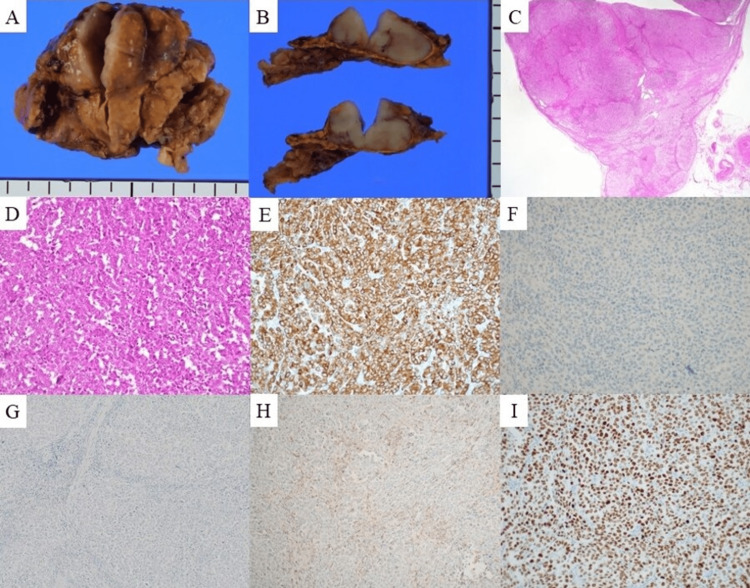
Pathological findings of the resected sample of adrenal gland. Pathological examination of sample (A) demonstrates smooth white cut surface (B) and poorly differentiated solid carcinoma (C). (D) Hematoxylin and eosin staining of the tumor sample. Immunohistochemistry staining revealed (E) CK 7 (+), (F) CK 20 (-), (G) inhibin (-), (H) napsin A (+), and (I) TTF-1 (+). CK: cytokeratin; TTF-1: thyroid transcription factor-1

The adrenal tumor was diagnosed as AM-CUP, and surgical resection was performed. Pathological examination by hematoxylin and eosin staining revealed a poorly differentiated solid carcinoma. Immunohistochemical staining revealed CK 7 (+), CK 20 (-), TTF-1 (+), inhibin (-), napsin A (+), prostate-specific antigen (PSA) (-), paired-box gene 8 (PAX8) (-), GATA3 (-), CDX-2 (-), synaptophysin (-), chromogranin (-), CD56 (-), and p40 (-) (Figure 4, panels A-I).

**Figure 5:**
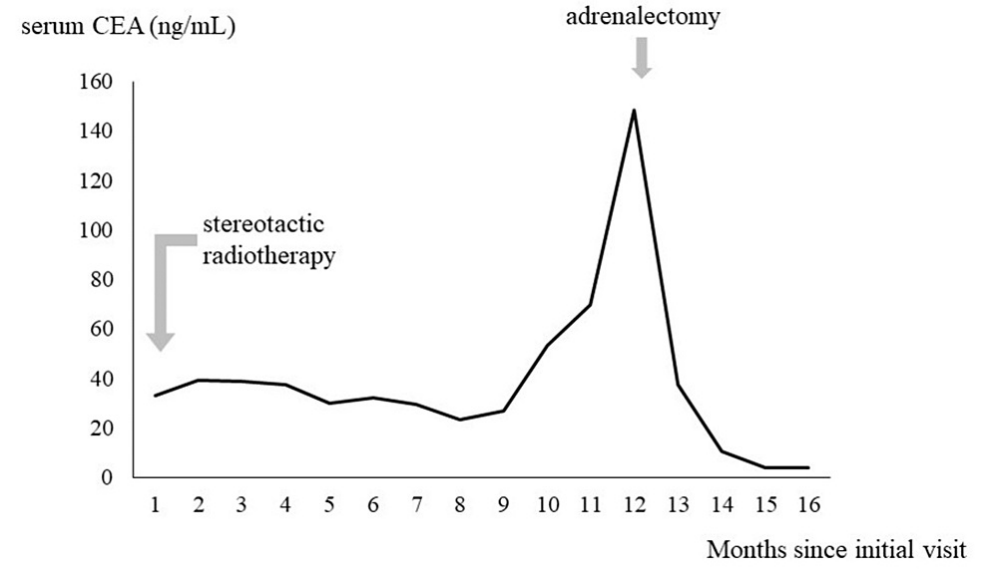
Clinical course of serum CEA level. Clinical course of serum CEA level and therapeutic event demonstrated elevation of serum CEA level during follow-up period and prompt normalization after adrenalectomy. CEA: carcinoembryonic antigen

After surgical resection of the adrenal tumor, the serum CEA level normalized (Figure 5). The postoperative course was uneventful, and the patient was discharged from our hospital seven days after surgery. No recurrence was confirmed over a two-year follow-up period. Written informed consent was obtained from the participant.

## Discussion

We report a rare case of BM and AM of unknown primary origin. In our case, the immunohistochemistry staining pattern suggested that the adrenal tumor was most likely a metastasis from lung adenocarcinoma, presenting TTF-1 positivity [8]. As the patient had a history of colon cancer, we first presumed that the BM and AM originated from the colon. However, BM and AM from colon cancer are relatively rare, and the staining pattern of previous colon cancer was inconsistent with that of the adrenal tumor [9-11]. Thus, we concluded that the metastasis was not from the colon. Contrarily, BM and AM are more common in lung cancers. Approximately 20-50% of BMs originate from lung cancer [2], and 20% of patients with lung cancer develop BM during their clinical course [12]. AM from lung cancer is not extremely rare, with a reported prevalence of 1.6-3.5% [13]. Thus, the BM and AM in our case possibly originated from lung cancer.

TTF-1 is overexpressed in lung and thyroid carcinomas and shows higher specificity in these two organs [14]. Thus, we suspected the origin of BM and AM to be thyroid carcinoma and lung cancer. However, BM and AM from thyroid carcinoma are not as common as those from lung cancer. The prevalence of BM from thyroid carcinoma is reported to range from 0.15% to 1.4% [15], and AM from thyroid carcinoma is considered uncommon [16]. Therefore, the possibility that the BM and AM originated from the thyroid was unacceptable. Further, napsin A was expressed in our case, which also supports the adrenal tumor originated from lung cancer [17].

The majority of adrenal metastases are reported to be bilateral, which is inconsistent with our case, and primary adrenocortical carcinoma (ACC) should be considered when unilateral adrenal tumors are detected [5]. However, primary ACC is uncommon, with a worldwide annual incidence of two per million [18]. Based on pathological examination, this tumor was diagnosed as metastasis rather than primary ACC. Elevated CEA levels are associated with several cancers, such as those of the colon, breast, lung, pancreas, liver, stomach, prostate, and thyroid, not including ACC [19]. This also supported our diagnosis that this tumor was not a primary ACC.

Regarding the treatment of BM and AM-CUP, several studies have reported that aggressive treatment, such as surgical resection and radiotherapy, which can be beneficial. Surgery and radiotherapy are reported to be effective for brain metastasis of undetected primary tumors, with a median overall survival rate of 13.4 months [3]. In our case, radiotherapy was performed because of damage to the visual cortex. The treatment was effective, and the symptoms were relieved and did not recur.

There is insufficient evidence suggesting that adrenalectomy for AM-CUP improves prognosis; however, a case report of AM-CUP presented an uneventful outcome [7]. Additionally, Sastry et al. reported that adrenalectomy for adrenal metastasis from lung cancer increased the long-term survival rate compared to chemotherapy or radiotherapy alone [20]. Given this evidence and for tissue diagnosis, we performed surgical resection of the AM-CUP, which proved beneficial.

In this case, we did not perform a biopsy from brain tumor, which was a limitation of this case report. Although the imaging of brain indicated that the brain tumor was metastasis and the tumor possibly originated from the same origin with adrenal metastasis, we could not conclude that the tumors came from the same origin. However, in that situation, our strategy was acceptable for benefit of the patient.

## Conclusions

In spite of close inspection of the primary tumor, the occult metastatic tumor in brain and adrenal gland was detected. CUP presents a challenge in patient care due to lack of information about the primary tumor. Despite its rare occurrence, the unilateral AM in our patient was diagnosed as AM from a primary lung tumor. Some cases, like our case with BM and AM, require aggressive treatment, including radiotherapy and surgical therapy. Moreover, detection of the primary origin requires several investigations to exclude different possibilities. Pathological and immunohistochemistry findings allowed us to reach an accurate diagnosis.

In our case, the patient’s symptoms and condition improved after radiotherapy and adrenalectomy. Thus, when metastatic lesions with unknown primary tumors are confirmed by pathological examination or even clinically diagnosed by imaging, clinicians should consider aggressive therapeutic options and investigation of the primary tumor site.
